# Storage stability of liposomes stored at elevated subzero temperatures in DMSO/sucrose mixtures

**DOI:** 10.1371/journal.pone.0199867

**Published:** 2018-07-05

**Authors:** Bulat Sydykov, Harriëtte Oldenhof, Harald Sieme, Willem F. Wolkers

**Affiliations:** 1 Institute of Multiphase Processes, Leibniz Universität Hannover, Hannover, Germany; 2 Unit for Reproductive Medicine, Clinic for Horses, University of Veterinary Medicine Hannover, Hannover, Germany; University of Maryland, UNITED STATES

## Abstract

Cryopreservation of biological materials is predominantly done using liquid nitrogen, and its application involves high maintenance costs and the need for periodical refilling of liquid nitrogen. Stable storage in mechanical freezers at −80°C would eliminate these issues and allow for shipment of frozen specimens using dry ice. In this work, the possibility of increasing the storage temperature of cryopreserved samples to −80°C by using combinations of DMSO and sucrose has been studied. Preservation efficacy was studied by measuring stability of liposomes encapsulated with carboxyfluorescein during storage at −150, −80 and −25°C for up to three months. Thermal and molecular mobility properties of the different DMSO-sucrose formulations were measured using differential scanning calorimetry, whereas hydrogen bonding interactions of the formulations were probed by Fourier transform infrared spectroscopy. It was found that addition of sucrose to DMSO solutions increases the T_g_, and decreases molecular mobility in the glassy state at a particular temperature. Although it was expected that storage above or close to T_g_ at −80°C would affect liposome stability, stability was found to be similar compared to that of samples stored at −150°C. Higher molecular mobility in the glassy state could not be associated with faster CF-leakage rates. Distinct differences in storage stability at −25°C, far above T_g_, were found among the sucrose/DMSO formulations, which were explained by the differences in permeability of sucrose and DMSO resulting in different levels of osmotic stress in the formulations.

## Introduction

Cryopreservation of biological materials allows for long term storage at subzero temperatures. Cryopreservation of mammalian cells requires use of membrane permeating cryoprotective agents (CPAs) allowing cells to survive freezing and thawing. Permeating cryoprotective agents like dimethyl sulfoxide (DMSO), glycerol, and ethylene or propylene glycol can move across cellular membranes and hence provide intracellular protection. They provide protection by stabilizing biomolecules and cellular structures, minimizing osmotic stress, and limiting the damaging effects of ice formation [1]. Furthermore, CPA addition facilitates formation of a glassy state in which material is embedded and preserved [2–5]. Long-term preservation is predominantly done using liquid nitrogen, either in the liquid nitrogen itself or above the vapor phase of the liquid at temperatures at or below −150°C [6]. Use of liquid nitrogen for storage, however, involves high maintenance costs and the need for periodical refilling [7–10]. If stable storage in mechanical freezers at −80°C would be feasible this would eliminate these issues, and allow for shipment of frozen specimens on dry ice [11].

Stable storage at elevated subzero temperatures requires development of cryopreservation formulations that ensure storage sufficiently below the glass transition temperature (T_g_). A glass is a metastable supercooled liquid state and has limited molecular mobility. To increase the T_g_ of formulations for use at −80°C, ordinarily used CPAs like DMSO can be combined with molecular compounds with high glass transition temperatures. Examples of such compounds are disaccharides (sucrose, trehalose) and polymers including ficoll, hydroxyethyl starch (HES) and polyvinyl pyrrolidone (PVP) [12–14]. Multiple reports exist in which such combinations were successfully employed for cryopreservation of mammalian cells and tissues [15–18]. Yuan et al [7] actually showed that supplementing DMSO formulations with ficoll allowed preservation of pluripotent stem cells for up to one year at −80°C, whereas formulations without ficoll were not effective. They discussed that adding ficoll particularly decreased the incidence of recrystallization during storage.

Freezing of a cryopreservation solution results in formation of an ice fraction, while the remaining non-frozen fraction vitrifies at low enough temperatures. The glassy state is a metastable state. Due to molecular mobility (i.e. motions) and the tendency to transition into the crystalline state, ice nucleation and crystal growth (i.e. recrystallization) may occur during storage. The rate at which relaxation occurs is dependent on the absolute temperature as well as the molecular compounds forming the glassy state [19]. Within a glass, different types of cooperative and localized motions can be distinguished, all with their characteristic relaxation times [20,21]. Relaxation rates in the glassy state have been correlated with chemical instability of biospecimens embedded in the glassy matrix [22–25]. High molecular motion propels protein aggregation [25], accumulation of oxidation products [24], and crystallization [26,27]. If disaccharides or polymers are added, this leads to stronger interactions between molecules, longer relaxation times and reduced molecular mobility (i.e. slowed chemical reactions), as well as resistance towards crystallization [28–30]. Molecular mobility in the vitrified or supercooled state can be evaluated using multiple methods, including dielectric spectroscopy [31,32], isothermal microcalorimetry [33], nuclear magnetic resonance [34] and differential scanning calorimetry [35–37].

The aim of the current study was to (1) evaluate effects of sucrose addition to DMSO cryopreservation formulations on the T_g_ and molecular mobility (i.e. structural relaxation) in the glassy state, and (2) correlate these physical characteristics with storage stability of cryopreserved liposomes stored at different subzero temperatures (below and above T_g_). Physical characteristics of glasses in the frozen state were analyzed using differential scanning calorimetry (DSC), whereas liposomes encapsulated with a fluorescent dye were used to study membrane leakiness during frozen storage. In addition, to obtain insights in molecular interactions between sucrose and DMSO, Fourier transform infrared spectroscopy (FTIR) was used.

## Materials and methods

### Differential scanning calorimetry (DSC) for determining glass transition temperatures and enthalpy relaxation

Various DMSO/sucrose mixtures were prepared in phosphate buffered saline (PBS; 137 mM NaCl, 2.7 mM KCl, 10 mM Na_2_HPO_4_, 1.8 mM KH_2_PO_4_, pH 7.4); DMSO concentrations ranged from 0–20% (v/v), and were prepared alone as well as combined with 0.5 or 1 M sucrose.

DSC measurements were done using a Netzsch 204F1 Phoenix instrument (Netzsch-Geraetebau GmbH, Selb, Germany). Calibration was performed using adamantane, bismuth, indium, zinc, selenium, and cesium chloride; according to the instructions provided by the manufacturer. An empty pan was used as a reference sample. For samples, approximately 10 μL (~10 mg) was added into a 25-μL aluminum pan, after which it was sealed hermetically. Glass transitions and melting behavior were studied by cooling samples to –150°C with 20°C∙min^–1^ followed by heating to 30°C at 10°C∙min^–1^, while monitoring the heat flow. In order to study enthalpy relaxation of glasses, samples were exposed to aging (i.e. storage at different temperatures for different durations) inside the DSC chamber. Therefore, using cooling and heating rates described above, samples were cooled to −150°C followed by heating to a specific temperature (varying from −115°C to −65°C) at which samples were maintained for up to 6 hours. After this, the sample was cooled again to −150°C and heated to 30°C. Thermal events were determined from the obtained thermograms, using Netzsch software.

Unless stated otherwise, the glass transition temperature (T_g_) was determined as the onset temperature of the temperature range where a change in specific heat occurred coinciding with the transition from glassy-to-liquid state. The change in heat flow at T_g_ divided by the heating rate is assigned ΔC_p_. Enthalpy relaxation (or enthalpy recovery) was observed as an endothermic event occurring superimposed on top of the glass transition. The storage-duration-dependent increase in the area of this event (ΔH_relaxation_) was determined by subtracting the thermogram of a sample measured directly after preparation from that of samples stored for different durations at a specific temperature below T_g_.

To derive parameters describing enthalpy relaxation and molecular mobility in a glass, plots were constructed in which ΔH_relaxation_ (in J g^−1^) was plotted versus the storage duration (t in s). Enthalpy relaxation during storage then can be fitted using the Kohlrausch-Williams-Watt (KWW) equation [38]. First, the maximum possible enthalpy recovery ΔH_∞_ at a given storage temperature is described by:

$${\Delta H}_{\infty }={\Delta C}_{\text{p}}(T_{g,midpoint}-T_{\text{storage}})$$ (1)

here ΔC_p_ (in J∙g^−1^∙K^−1^) is the change in heat capacity at T_g_midpoint_ and T_storage_ (both in K) represents the storage temperature. To take into account the differences in broadness of the glass transitions, the midpoint T_g_ was taken. Then, ΔH_relaxation_ can be described as a function of ΔH_∞_, the mean relaxation time τ (in s) and a non-exponential parameter describing the distribution of relaxation times (β):

$$\Delta H_{\text{relaxation}}=\Delta H_{\infty }\left\{1-exp\left[-{\left(\frac{t}{\tau }\right)}^{\beta }\right]\right\}$$ (2)

β is 1 in case of a single relaxation time, whereas smaller values (≤1, ≥0) are indicative for a wider distribution of τ. For determining the activation energy E_a_ (in kJ∙mol^–1^), Arrhenius plots on the relaxation time versus storage temperature can be created and fitted using:

$$\text{ln}\tau =\frac{E_{a}}{R}\times \frac{1000}{T_{\text{storage}}}+\text{ln}\tau_{0}$$ (3)

here R is the gas constant (8.314 J∙mol^–1^∙K^–1^), the mean relaxation time τ is given in s and storage temperature T_storage_ in K.

### Assessment of storage stability of cryopreserved liposomes with entrapped carboxyfluorescein

Liposomes with trapped carboxyfluorescein (CF) were prepared as previously described [39,40], with minor modifications [41]. A solution containing 20 mg∙mL^−1^ egg phosphatidylcholine (PC), 10 mM HEPES pH 7.5 and 100 mM CF was extruded through polycarbonate membrane filters with 100 nm pores (Whatman, Maidstone, Kent, UK) using an Avanti mini-extruder (Avanti Polar Lipids, Alabaster, AL, USA). Liposomes with entrapped CF were separated from free (i.e. extraliposomal) CF via passage of the solution obtained after extrusion through a Sephadex G50 column, using 10 mM HEPES pH 7.5 as eluent.

For cryopreservation, liposomes were diluted by adding an equal volume of DMSO/sucrose solution or 10 mM HEPES as a control (10 μL each). Final CPA concentrations that were tested were: (S1) 5% DMSO, 1 M sucrose; (S2) 10% DMSO, 1 M sucrose; (S3) 5% DMSO, 0.5 M sucrose; and (S4) 10% v/v DMSO, 0.5 M sucrose. Samples were frozen via plunging microtubes in liquid nitrogen, after which they were transferred to mechanical freezers of −150 or −80°C (Sanyo Electric Biomedical Co., Bad Nenndorf, Germany) or a −25°C fridge, for storage for up to 3 months. Samples were thawed after different storage durations via incubation at room temperature, and analyzed directly after thawing.

CF-retention was measured by diluting liposome samples with 3 mL 10 mM HEPES pH 7.5. CF fluorescence is quenched at high concentrations as initially present inside the liposomes, and increases upon (dilution and) leakage into the medium. Maximum fluorescence levels after complete leakage were determined by adding 60 μL 1% (v/v) Triton-X100. Fluorescence measurements were done using a Perkin-Elmer LS55 spectrofluorometer (PerkinElmer, Norwalk, CT, USA) with excitation and emission wavelengths set at respectively 460 and 550 nm (both with 2.5 nm slit width), using a 5 s integration time. The percentage of CF-retention inside liposomes after different treatments was calculated by comparing the initial fluorescence values (assessed directly after preparing CF containing liposomes) with the values after addition of CPAs (pre-freeze), or after cryopreservation, storage and thawing:

$${CF}_{\text{retention}}=(\frac{d\times F_{\text{B}}-F_{\text{A}}}{d\times F_{\text{b}}-F_{\text{a}}})\times (\frac{F_{\text{b}}}{F_{\text{B}}})\times 100\%$$ (4)

here F_A_ and F_B_ represent the CF fluorescence readings of treated samples before and after addition of Triton-X100, respectively. F_a_ and F_b_ are the reference fluorescence values of the CF-liposomes without further treatment, before and after addition of Triton-X100. Factor d refers to the dilution factor for F_B_ and F_b_, due to addition of Triton-X100, and factor F_b_/F_B_ takes into account losses of CF after processing. For analysis of storage stability, plots on CF-retention versus the storage duration (t_storage_) were fitted using:

$$\text{ln}({CF}_{\text{retention}})=-k\times t_{\text{storage}}+C$$ (5)

here k is the degradation or leakage rate, and C a constant. Statistical analyses of CF-retention values were performed using SigmaPlot v13 (Systat Software GmbH, Erkrath, Germany). Comparison between CF-retention at days 1 and 90 for specific formulation was done using paired T-tests with one-sided P-value, whereas one way ANOVA was used to compare CF-retention between formulations at specific time points. For visualization of effects of the glass transition temperature, leakage rates in turn can be plotted as a function of (T_storage_-T_g_).

### Fourier transform infrared spectroscopic (FTIR) analysis of hydrogen bonding interactions in DMSO/sucrose solutions

Infrared spectra of DMSO/sucrose solutions, prepared as described above, were recorded using a Perkin-Elmer 100 Fourier transform infrared spectrometer (PerkinElmer, Norwalk, CT, USA) equipped with an attenuated total reflection (ATR) accessory with diamond/ZnSe crystal. Five-μL solution was added onto the ATR crystal and spectra were collected at room temperature, using an automatic CO_2_/H_2_O vapor correction algorithm. Acquisition parameters were: 4 cm^–1^ resolution, 8 co-added interferograms, and 4000–650 cm^–1^ wavenumber range.

DMSO-dependent changes in relative contributions of different hydrogen bonding interactions were characterized as described in detail elsewhere [42]. In short, the OH-stretching vibration band (νOH) at ~3600−3000 cm^−1^ was studied for sucrose solutions (0.5 or 1 M) with increasing DMSO concentrations (0–20%). νOH was fitted using multiple Gaussian profiles, with peaks at 3139 cm^−1^ (representing fully hydrogen bonded water), 3241 cm^−1^ (symmetrically hydrogen bonded water), 3389 cm^−1^ (asymmetrically hydrogen bonded water), and 3533 cm^−1^ (weakly hydrogen bonded water). Fitting was done using PerkinElmer and Omnic software (Thermo Electron Corporation, Waltham, MA, USA).

## Results

### Glass transition temperatures and enthalpy relaxation

**Fig 1:**
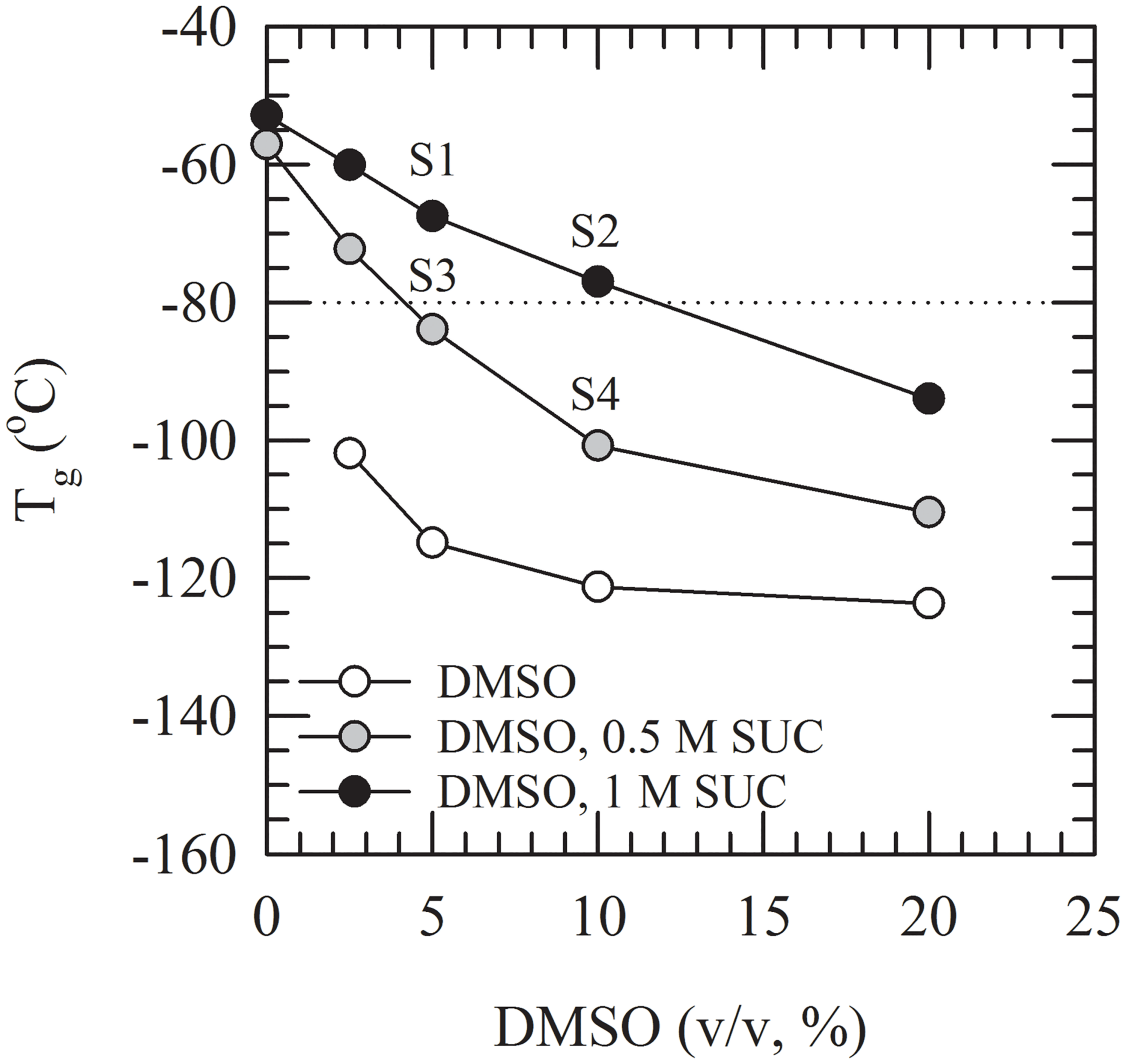
Glass transition temperatures of solutions composed of different contents of DMSO and sucrose. The onset temperature of glass transition was determined using DSC. This was done for 0–20% (v/v) DMSO solutions without supplements (white circles), as well as supplemented with 0.5 M sucrose (grey circles) or 1 M sucrose (black circles). For liposome storage experiments, performed at −80°C (dotted line), four different formulations were selected (labeled S1–4). Solution S1 was composed of 5% DMSO with 1 M sucrose (T_g_: −68°C), S2 of 10% DMSO with 1 M sucrose (T_g_: −77°C), S3 of 5% DMSO with 0.5 M sucrose (T_g_: −84°C) and S4 of 10% DMSO with 0.5 M sucrose (T_g_: −101°C).

DSC was used to measure the glass transition temperatures of DMSO/sucrose freeze-concentrated solutions (Fig 1). S1 File contains the minimal data set of all Figures presented in this manuscript. Presence of ice was evident as an exothermic/endothermic event during cooling/heating (data not shown). The T_g_ of 0.5 and 1 M sucrose solutions without DMSO were determined to be −57 and −52°C, respectively. Addition of DMSO has a plasticizing effect, which is evident as a decrease in T_g_ with increasing DMSO contents added to the sucrose solutions. In case of 0.5 M sucrose this effect is more pronounced and T_g_ decreases to a greater extent (i.e. larger decrease with increasing DMSO concentration) as compared to 1 M sucrose.

For liposome cryopreservation studies, and assessments on storage stability at elevated subzero temperatures including −80°C, four different formulations were selected. DMSO/sucrose solutions were chosen that had values of T_g_ close to −80°C. Solutions included: (S1) containing 5% DMSO and 1 M sucrose with a T_g_ of −68°C, (S2) containing 10% DMSO and 1 M sucrose with a T_g_ of −77°C, (S3) containing 5% DMSO and 0.5 M sucrose with a T_g_ of −84°C, and (S4) containing 10% DMSO and 0.5 M sucrose with a T_g_, of −101°C.

**Fig 2:**
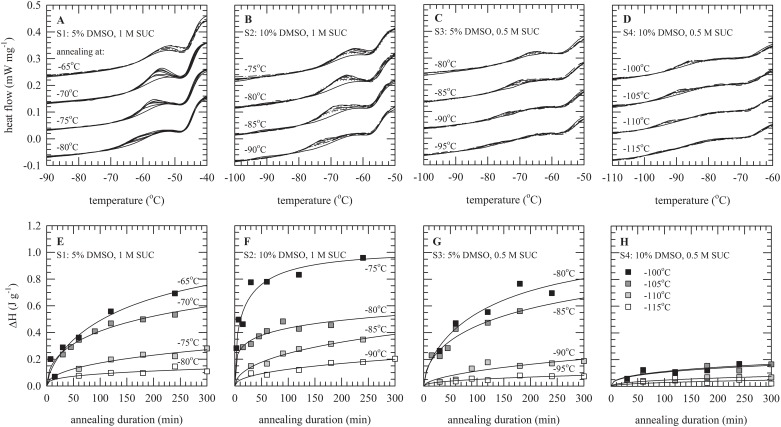
Enthalpy relaxation behavior of DMSO/sucrose solutions (S1–4) at different temperatures below the T_g_ of the solutions. DSC pans with solution were maintained ~0–15°C below T_g_ for up to 300 min, after which thermograms were recorded (A–D). Enthalpy relaxation is evident as an endothermic event (oriented upwards) on top of the glass transition event, increasing with storage duration while decreasing if further from T_g_. The area of this event, ΔH_relaxation_, was determined and plotted versus the storage duration (E–G), for the indicated storage temperatures. Solution S1 was composed of 5% v/v DMSO, 1 M sucrose (A,E), S2 of 10% v/v DMSO, 1 M sucrose (B,F), S3 of 5% DMSO, 0.5 M sucrose (C,G), and S4 of 10% v/v DMSO with 0.5 M sucrose D,H).

DSC thermograms illustrating enthalpy relaxation behavior of these formulations, during storage at four different temperatures just below their T_g_, are shown in Fig 2A–2D. Here it can be seen that the area of the endothermic event on the glass transition (i.e. enthalpy relaxation) increases as a function of the storage time, whereas this is more pronounced in case of storage closer to T_g_. In addition, the temperature at which enthalpy relaxation is observed shifts to higher temperatures with increasing storage temperature. As described in detail above, ΔH_relaxation_ was normalized to ΔH_∞_ and plotted as a function of storage duration. In Fig 2E–2H it can be clearly seen that there are distinct differences amongst S1–4 in the temperature and storage duration dependent changes in enthalpy relaxation (i.e. ΔH_relaxation_/ΔH_∞_). It should be noted that also the absolute temperature affects enthalpy relaxation kinetics. Enthalpy relaxation values for formulation S4 stored for 6 h 10°C below T_g_ at −100°C, do not exceed 0.2, whereas values above 0.6 were found for the other formulations if 10°C below T_g_ ranged from −65 to −80°C.

**Fig 3:**
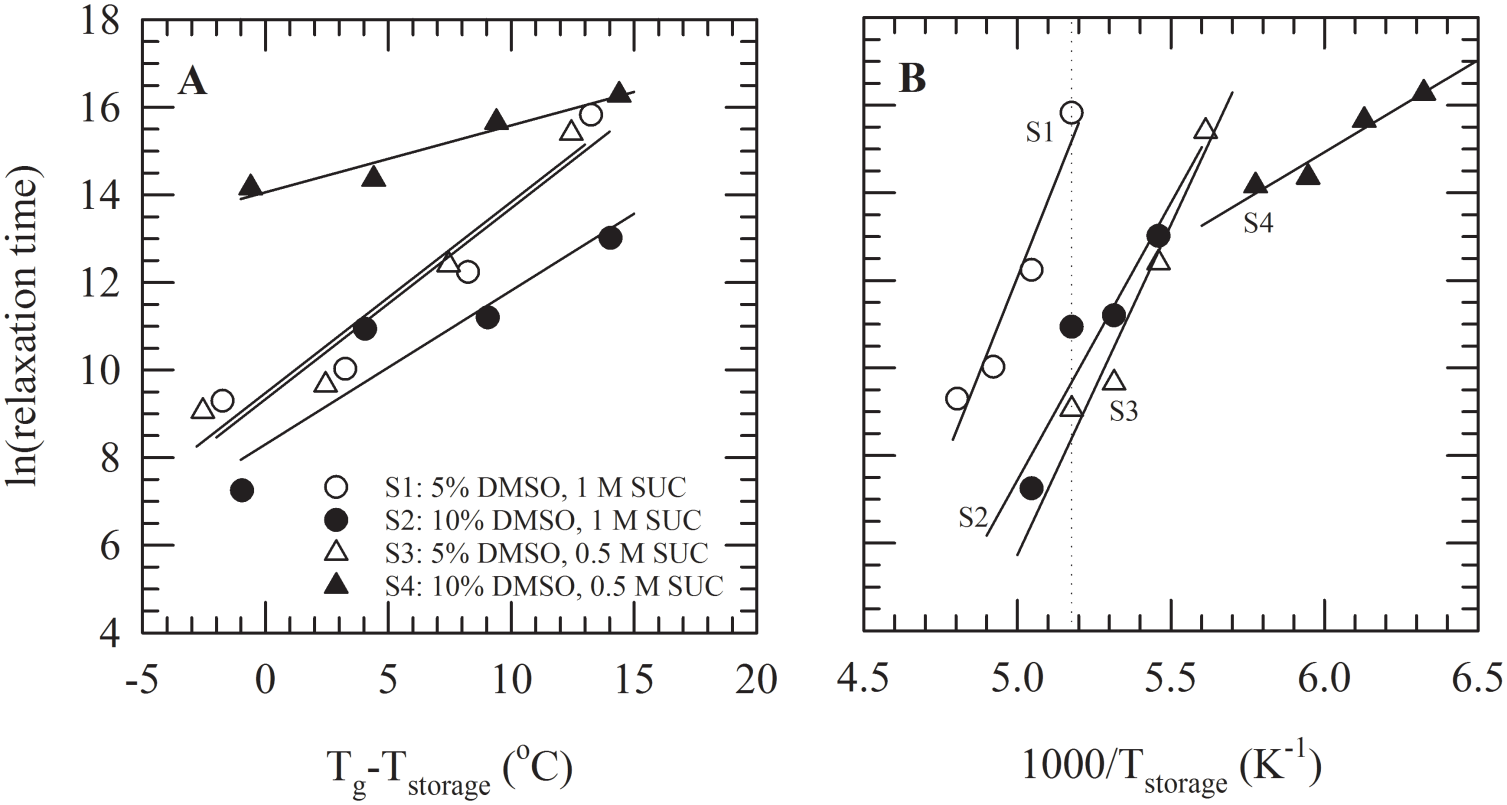
Parameters describing molecular mobility in glasses were determined by fitting DSC data on enthalpy relaxation versus storage. This was done for solution S1 (5% v/v DMSO, 1 M sucrose, white circles), S2 (10% v/v DMSO, 1 M sucrose, black circles), S3 (5% v/v DMSO with 0.5 M sucrose, white triangles) and S4 (10% v/v DMSO with 0.5 M sucrose, black triangles). In panel A, for S1–4, the natural logarithm of the relaxation time is plotted versus the difference between the storage temperature and T_g_. In the panel B, data are presented in Arrhenius plots for deriving activation energies. The dotted line indicates −80°C.

Plots of enthalpy relaxation versus storage time at different temperatures were fitted using the KWW equation to derive the mean relaxation time τ, and parameter β describing the distribution of relaxation times. Fig 3A shows the linear dependence of the natural logarithm of τ as function of the storage temperature with respect to T_g_. Higher values for ln(τ) are indicative for lower molecular mobility. Furthermore, lower values of dln(τ)/dT (i.e. the slope) indicate a smaller change in molecular mobility with increasing temperature. For all formulations, τ increases (i.e. molecular mobility decreases) with storage at temperatures further below T_g_. For storage at temperatures close to T_g_, τ-values are lowest for solution S2 and highest for S4. Solution S4 exhibits the lowest dln(τ)/dT-value, however, this formulation had the lowest T_g_ and consequently storage was done at the lowest absolute temperatures.

Arrhenius plots were constructed to compare τ-values at absolute temperatures and to derive activation energy values for S1–4 (Fig 3B). Activation energies were: 147 kJ·mol^−1^ for S1, 105 kJ·mol^−1^ for S2, 126 kJ·mol^−1^ for S3, and 35 kJ·mol^−1^ for S4. Furthermore, via extrapolation, relaxation times at −80°C were estimated. These were: 124116 min (i.e. 2069 h or 86 d) for S1, 940 min (i.e. 16 h) for S2 and 143 min (i.e. 2.4 h) for S3. No relaxation time was determined for S4, since −80°C is above the T_g_ of this formulation. The distribution of relaxation times, β, at −80°C was determined to be 0.33±0.18 for S1, 0.25±0.06 for S2 and 0.66±0.14 for S3.

### Storage stability of cryopreserved liposomes

**Fig 4:**
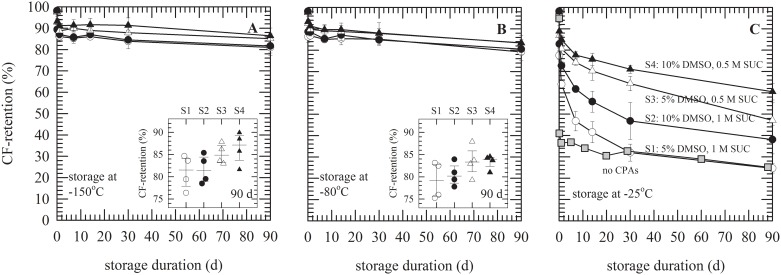
Storage stability of PC liposomes with trapped CF, frozen in DMSO/sucrose solutions and stored at different temperatures. Cryoprotective solutions tested were: S1 (5% DMSO, 1 M sucrose, white circles), S2 (10% DMSO, 1 M sucrose, black circles), S3 (5% DMSO with 0.5 M sucrose, white triangles), and S4 (10% DMSO with 0.5 M sucrose, black triangles) HEPES buffered solution without further supplements (grey squares) served as a control. Samples were stored at −150°C (A), −80°C (B) and −25°C (C) for up to 3 months. As a measure for storage stability, CF-retention (i.e. protection against membrane leakiness) was assessed and plotted versus the storage duration. The insets in the panels (A) and (B) show the CF-retention after 90 d at −150°C and −80°C, respectively (no significant differences in CF-retention were found among the formulations). Data points representing mean values ± standard deviations were calculated from four measurements (control samples were measured once).

**Fig 5:**
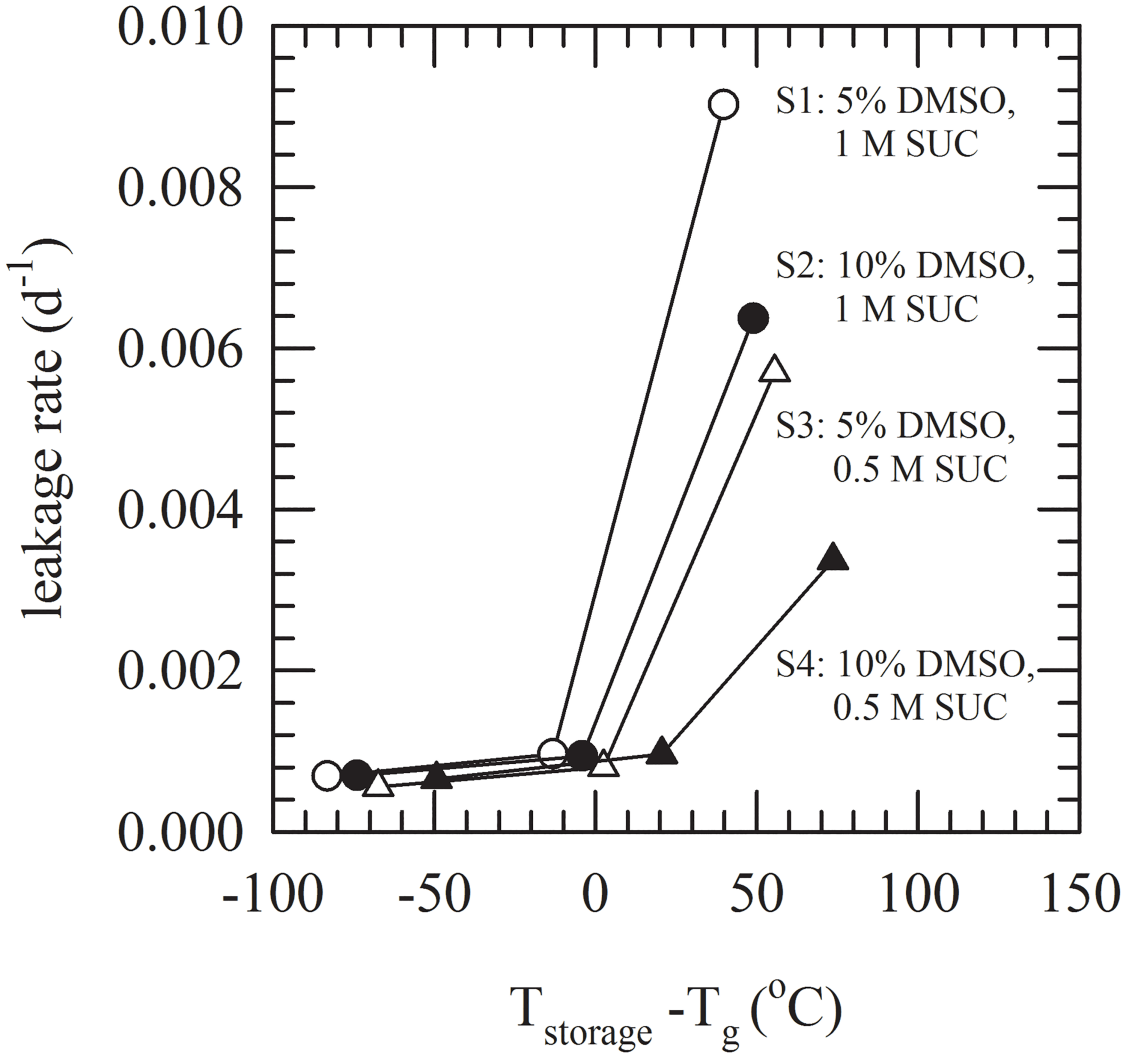
CF leakage rates of PC liposomes with trapped CF in DMSO/sucrose solutions at different subzero temperatures. The difference between the storage temperature and T_g_ was plotted against the CF leakage rate. Cryoprotective solutions that were tested: S1 (5% DMSO, 1 M sucrose, white circles), S2 (10% DMSO, 1 M sucrose, black circles), S3 (5% DMSO with 0.5 M sucrose, white triangles), and S4 (10% DMSO with 0.5 M sucrose, black triangles).

The DMSO/sucrose formulations characterized above were used for cryopreservation of liposomes and to study their storage stability. CF-leakage from liposomes composed of PC with trapped CF was determined, after thawing, during storage for up to 3 months at different subzero temperatures (i.e. below and above −80°C as well as T_g_ of the respective formulations). CF-retention percentages were determined and plotted as a function of the storage duration (Fig 4). Maintenance of high CF-retention values is indicative for good liposome stability (i.e. minimal leakage). Prior to freezing, CF-retention was about 98%, irrespective of the DMSO/sucrose formulation added. If exposed to freezing and thawing and storage, CF-retention was dependent on the formulation and duration of storage. If stored at −150°C, there is a statistically significant decrease of the CF-retention for each formulation with values ranging from 83–94% at d 1 to 76–90% at d 90 (Fig 4A). However, differences amongst formulations after 3 months storage are not significant (inset of Fig 4A). Also at −80°C, there is a significant decrease in CF-retention for each formulation decreasing from 85–95% at d 1 to 75–88% after 3 months (Fig 4B). However, no significant differences were observed at d 90 among the formulations irrespective if storage was below or above T_g_ (inset of Fig 4B). Despite the fact that CF-retention after 3 months was slightly reduced compared to samples stored at −150°C for all formulations, differences were not significant. In case of storage at −25°C clear differences can be observed among the formulations S1–4 (Fig 4C). In the absence of CPAs, CF-retention decreases down to ~37% within 1 d storage. Higher CF-retention values were found with addition of DMSO/sucrose, namely 64±2 with S1 (5% DMSO, 1 M sucrose), 73±2 with S2 (10% DMSO, 1 M sucrose), 81±3 with S3 (5% DMSO, 0.5 M sucrose) and 85±2 with S4 (10% DMSO, 0.5 M sucrose). Here CF-retention decreased to values ranging from 25–61% after 3 months storage. Stability during storage was evaluated via determining leakage rates, from the slope in plots of CF-retention versus storage duration (d 1–90). This in turn was plotted as a function of the storage temperature with respect to T_g_ of the formulation (T_storage_−T_g_). In Fig 5 it can be seen that the leakage rate is minimal if specimens are stored below or even close to their T_g_. For storage at −25°C, which is far above T_g_ for all formulations, leakage rates are drastically increased. Leakage rates were found to be dependent on the formulation and decreased in the order S1 (0.0090 d^−1^), S2 (0.0064 d^−1^), S3 (0.0057 d^−1^), and S4 (0.0034 d^−1^).

### Hydrogen bonding interactions in DMSO/sucrose solutions

**Fig 6:**
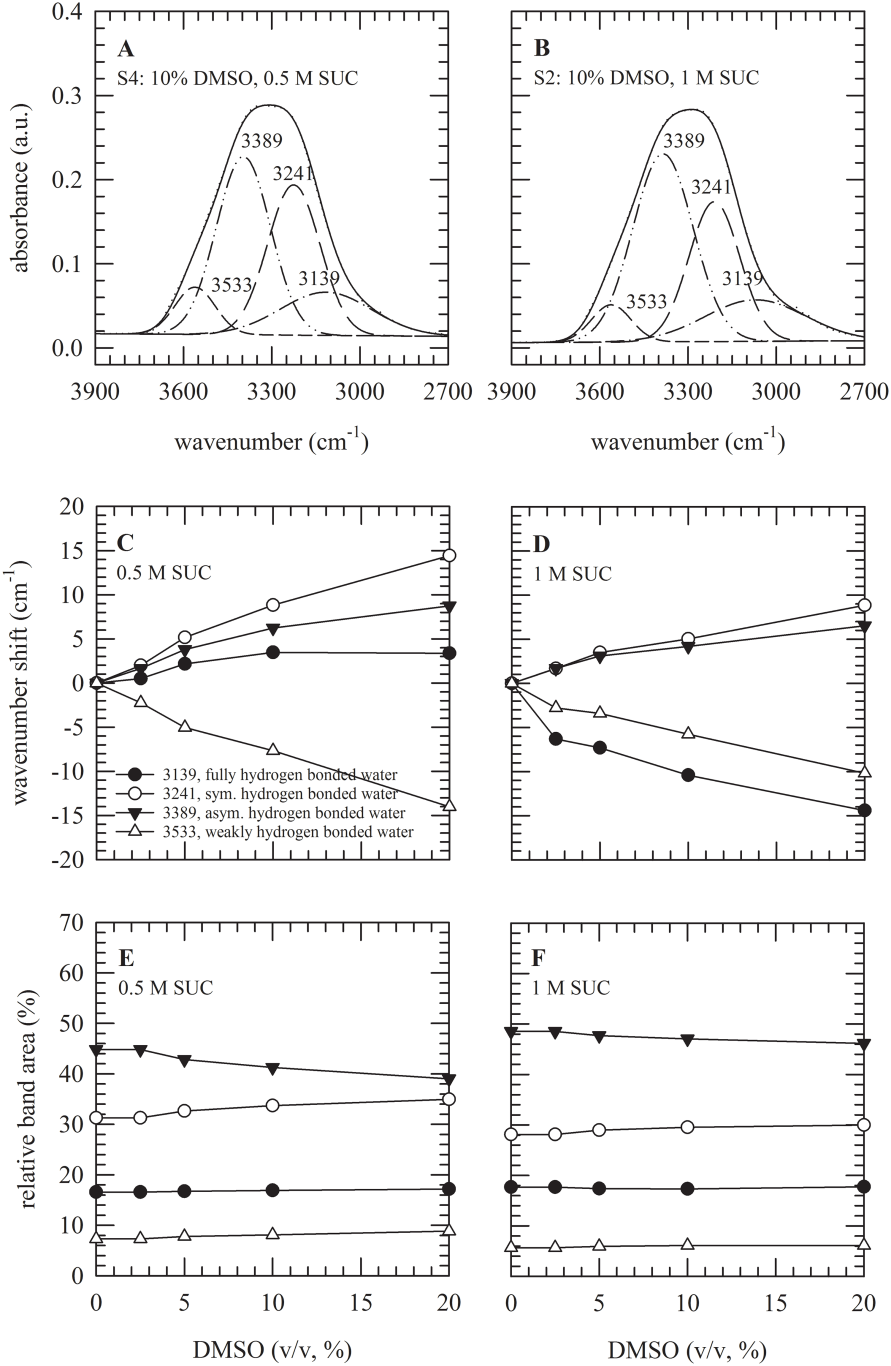
Water fraction distributions in solutions composed of DMSO and sucrose, determined from the OH-stretching band in FTIR spectra. In panel A–B, the 3900–2700 cm^−1^ spectral region is shown for two DMSO/sucrose solutions (S4: 10% v/v DMSO with 0.5 M sucrose, S2: 10% v/v DMSO with 1 M sucrose). Contributions of different water fractions were fitted, as Gaussian profiles centered initially at 3139 cm^−1^ (fully hydrogen bonded water), 3241 cm^−1^ (symmetrically hydrogen bonded water), 3389 cm^−1^ (asymmetrically hydrogen bonded water) and 3533 cm^−1^ (weakly hydrogen bonded water). The relative shifts in peak positions (C,D) and relative band areas (E,F) of these contributions were determined as a function of the DMSO concentration, in combination with either 0.5 M sucrose (C,E) or 1 M sucrose (D,F).

In order to study possible differences in hydrogen bonding interactions between S1–4, ATR-FTIR spectra were recorded at room temperature. Fig 6A and 6B shows examples to illustrate how curve-fitting of the OH-stretching bands of the DMSO/sucrose solutions was done. Gaussian profiles were used to resolve relative contributions of different types of hydrogen bonding interactions. Four different bands can be identified in the OH stretching region, reflecting different vibrational modes of H_2_O and different hydrogen bonding populations, namely weakly hydrogen-bonded water (~3533 cm^−1^), asymmetrically (~3389 cm^−1^) and symmetrically (~3241 cm^−1^) hydrogen-bonded water, and fully hydrogen-bonded water (~3139 cm^−1^). Adding DMSO to sucrose solutions results in changes in the peak positions of these hydrogen bonding populations reflecting DMSO-induced changes in the strength of hydrogen bonding interactions (Fig 6C and 6D) of water-sucrose solutions. With increasing DMSO concentrations, peak positions denoting symmetrically and asymmetrically hydrogen bonded water shift to higher wavenumbers indicating less strong hydrogen bonding interactions, whereas the weakly hydrogen bonded water peak shifts to lower wavenumbers. The latter indicates that more water molecules are participating in interactions with DMSO as compared to forming hydrogen bonds with each other. Similar behavior was seen for 0.5 and 1 M sucrose. However, DMSO addition differently acted on fully hydrogen bonded water; resulting in respectively an increase and decrease in the strength of interactions. Inspection of the relative contributions (i.e. band areas) of the different hydrogen bonding populations shows that the relative contribution of the population comprising asymmetrically hydrogen bonded interactions is larger in case of 1 M sucrose solutions. In addition, it decreases with increasing DMSO concentrations added to both 0.5 and 1 M sucrose. Contributions from symmetrically hydrogen bonded interactions and weakly hydrogen bonded water increase in both cases.

**Fig 7:**
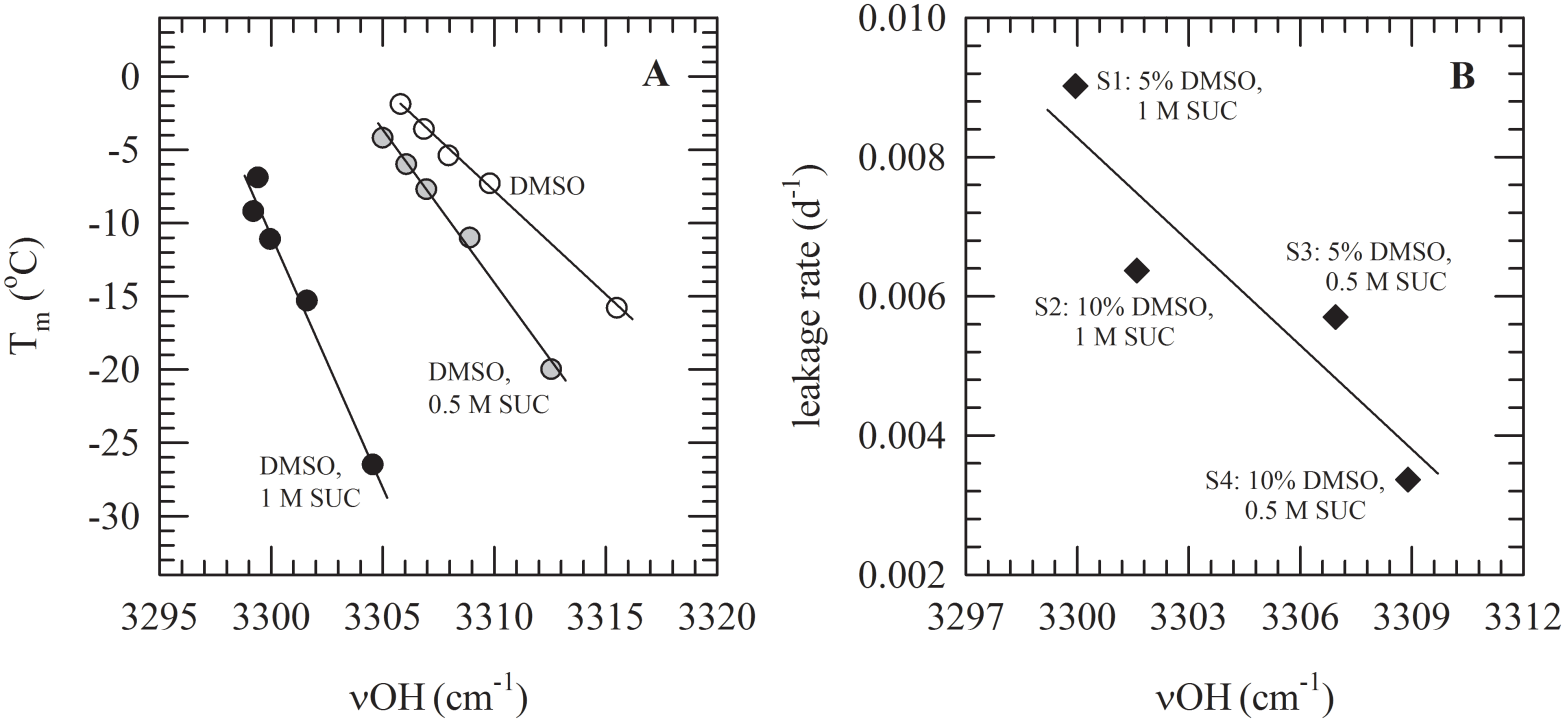
Correlation between thermal and stability parameters of PC liposomes with trapped CF in DMSO/sucrose solutions with the band position of the OH stretching band (νOH) of the protective solutions. In panel A, the onset temperatures of melting for DMSO solutions without supplements (white circles), with 0.5 M sucrose (grey circles) and 1 M sucrose (black circles) are plotted against the positions of νOH. In panel B, leakage rates determined for liposomes frozen with the selected formulations of DMSO and sucrose; S1 (5% DMSO, 1 M sucrose), S2 (10% DMSO, 1 M sucrose), S3 (5% DMSO with 0.5 M sucrose), and S4 (10% DMSO with 0.5 M sucrose) during storage at −25°C are plotted against νOH. νOH was determined at room temperature.

A correlation was found between the onset temperature of melting of the DMSO/sucrose formulations and the band position of the OH stretching band of the formulation (Fig 7A). Moreover, CF-leakage rates of liposomes frozen with formulations S1–4 that were stored at −25°C show a negative linear correlation with the band position of the OH stretching band (Fig 7B).

## Discussion

In this study different DMSO/sucrose formulations were used for cryopreservation of liposomes and to study preservation (i.e. storage stability) at different subzero storage temperatures. Formulations have been characterized in terms of their glass transition temperature and enthalpy relaxation behavior as a measure for molecular mobility in the glassy state. It was postulated that storage sufficiently below T_g_ allows little enthalpy relaxation thus increasing storage stability. It was found that addition of sucrose to DMSO formulations increases the T_g_ of the freeze concentrated solution and decreases molecular mobility in the glassy state at a particular temperature. Liposomes with entrapped CF were used as a model system to study protection against membrane leakage in different DMSO/sucrose formulations. All DMSO/sucrose formulations that were tested here were shown to have cryoprotective properties. CF-leakage rates were found to be negligible low during 3-months storage at −80°C irrespective whether the formulations were in a glassy state or not.

During storage at −150°C there is a small but significant decrease in CF-retention with very low leakage rates during storage for up to 3 months, irrespective of the formulation used. Storage at −150°C is at least 50°C below T_g_ for all formulations tested with structural relaxation times of several years, which implies that molecular mobility is practically ceased and hence damaging reactions are virtually absent [35]. Differences amongst formulations were expected during storage at −80°C either due to absence of a glassy state (10% DMSO/0.5 M sucrose) or increased molecular mobility (i.e. decreased relaxation times) in the glassy state at this temperature (5% DMSO/1 M sucrose>10% DMSO/1 M sucrose>5% DMSO/0.5 M sucrose). However, liposome stability at −80°C was found to be similar compared to that at −150°C, with no significant differences among the formulations.

The unexpected good stability of the formulation with 10% DMSO/0.5 M sucrose during storage at −80°C could be due to the broadness of the transition from the glassy to liquid state. The heterogeneity of the glass can also affect the derived molecular mobility parameters. The glass transition temperatures that are reported reflect the onset of the glass transition, i.e. the beginning of the transition from the glassy to liquid state. The difference between the endpoint of the glass transition and onset, i.e. the width of the glass transition, is higher in formulations with more DMSO or less sucrose. Formulation S4 with 10% DMSO and 0.5 M sucrose displays a broad glass transition with an onset at −101°C and an endpoint of −82°C, which is just 2°C below the storage temperature at -80°C. The broadness of the transition causes some parts of the glass to relax more slowly than other parts. Furthermore, also temperature itself is a protective factor, which could mask differences in leakage rates among the formulations despite differences in T_g_. Moreover, differences may become apparent after longer storage times. Inspection of the Arrhenius behavior of relaxation times in the glassy state revealed that the activation energy of the 10% DMSO/0.5 M sucrose formulation is clearly smaller compared to that of the other formulations, which were all in the same range. Higher E_a_-values have been associated with greater stability of glasses [14].

The finding that structural relaxation and molecular motion in the glassy state did not correlate with liposome stability can likely be explained by the fact that parameters derived here represent averaged of global and local mobilities [43]. Furthermore, the KWW model does not take the non-linearity of τ during storage into account [36,44]. Application of other models including the Adam-Gibbs model may provide additional insights [45,46]. However, this requires knowledge of the heat capacity above T_g_, which cannot be easily determined in our case because the samples directly start to melt after passing T_g_.

Inspection of hydrogen bonding interactions in DMSO/sucrose formulations in the liquid state revealed that higher DMSO concentrations are associated with a decreased strength of hydrogen bonding interactions, whereas higher sucrose concentration resulted in stronger interactions. If this behavior could be extrapolated to frozen storage above T_g_, it would be expected that molecular motion and therewith tendency for crystallization is slowest for formulations containing relatively high sucrose contents combined with low DMSO concentrations are the most stable [28], which was found not to be the case.

Storage above T_g_ at −25°C resulted in leakage during storage in all formulations. The progressive increase in leakage with storage duration may be related to ice crystal growth (i.e. devitrification) and osmotic stress. Ice crystals may cause mechanical stresses causing membrane rupture of the liposomes. Furthermore, the increased solute concentration in the unfrozen fraction may expose liposomes to osmotic stress causing dehydration-induced membrane phase changes and associated leakage [47,48]. At −25°C, liposome leakage rates were found to be higher in the presence of 1 M sucrose compared to those in the presence of 0.5 M sucrose, whereas 10% DMSO is preferred over 5% DMSO in terms of liposome leakage. Higher sucrose concentrations cause more osmotic stress due to the osmotic imbalance between the intra- and the extra-liposomal environment and the fact that sucrose is membrane impermeable. Since DMSO is a permeating agent, higher intraliposomal DMSO contents in turn may reduce osmotic forces and stress.

## Conclusion

Addition of sucrose to DMSO solutions increases the T_g_ and decreases molecular mobility in the glassy state at a particular temperature. Although it was expected that storage of liposomes with entrapped CF at −80°C, which is above or close to T_g_ for the DMSO/sucrose formulations studied here, would affect liposome stability, leakage rates were found to be the same in the different formulations and similar compared to those at −150°C. Moreover, higher molecular mobility in the glassy state (i.e. structural relaxation) did not result in higher leakage rates. Distinct differences in storage stability, i.e. CF-leakage, at −25°C, far above T_g_, were found among the sucrose/DMSO formulations, which likely can be attributed to the differences in membrane permeability of sucrose and DMSO resulting in different levels of osmotic stress in the formulations.

## Supporting information

S1 File Minimal data set of presented figures. (XLSX)
